# Concordance of Cataract Biometry Measurements Obtained via Swept-Source Optical Coherence Tomography Versus Optical Biometry

**DOI:** 10.3390/bioengineering13080874

**Published:** 2026-07-28

**Authors:** Ramadhan Ahmed, Kamini Narendra Reddy, Matthew Zeng, Mahad Mohamed, Nakul Shekhawat

**Affiliations:** 1 School of Medicine, Johns Hopkins University, Baltimore, MD 21205, USA; rahmed21@jh.edu (R.A.); mmoham36@jh.edu (M.M.); 2 Cornea Division, Wilmer Eye Institute, Johns Hopkins University School of Medicine, 600 North Wolfe Street, Wilmer/Woods 376, Baltimore, MD 21205, USA; kreddy13@jh.edu; 3 Department of Biology, Krieger School of Arts and Sciences, Johns Hopkins University, Baltimore, MD 21218, USA; xzeng27@jhu.edu

**Keywords:** Anterion, IOLMaster 700, SS-OCT, interdevice agreement, ocular biometry, intraocular lens selection, cataract surgery planning

## Abstract

**Purpose**: To examine concordance in biometry, keratometry, and intraocular lens (IOL) calculation between the Heidelberg Anterion Cataract App and the Zeiss IOLMaster 700 in a US population presenting for cataract evaluation. **Methods**: In this retrospective cross-sectional study, 64 eyes of 40 patients presenting for cataract evaluation underwent imaging with both devices. Agreement was assessed for axial length (AL), anterior chamber depth (ACD), lens thickness (LT), white-to-white distance (WTW), central corneal thickness (CCT), pupil diameter (PD), and anterior, posterior, and total keratometry using Lin’s concordance correlation coefficient (CCC), Bland–Altman analysis and generalized estimating equations. Non-toric and toric IOL calculations were performed with the Barrett Universal II and Barrett True-K Toric formulas, respectively. **Results**: Concordance was excellent for AL, ACD, LT, and CCT (CCC > 0.980), with no significant difference in AL. Compared with IOLMaster 700, Anterion measured larger PD (+0.56 mm), deeper ACD (+0.07 mm), thicker LT (+0.07 mm), thinner CCT (−3.19 μm), and smaller WTW (−0.22 mm; all *p* < 0.01). Anterior keratometry showed excellent concordance (CCC ≥ 0.980) despite small flatter offsets in average K, K1, K2 and difference in (Δ)K (−0.13, −0.08, −0.16 D, −0.10). Concordance was poor for posterior keratometry (average K: CCC = 0.529; Anterion steeper by 0.34 D), fairly good for total keratometry (average K: CCC = 0.891, Anterion flatter by 0.68 D), and these systematic inter-device offsets may be clinically relevant. For non-toric calculations, unrounded spherical IOL power agreed within 0.50 D in 88.88% (N = 56/63) of eyes and predicted residual refractive error within 0.25 D in 95.24% (N = 60/63). For toric calculations, predicted residual refractive error and residual astigmatism agreed within 0.25 D in 95.16% (N = 59/62) and 85.48% (N = 53/62) of eyes, respectively. **Conclusions**: Anterion and IOLMaster 700 showed strong agreement in core biometry, anterior keratometry, and predicted refractive outcomes, though posterior and total keratometry differed systematically. Postoperative refractive outcome studies are needed before the devices can be considered interchangeable in routine practice.

## 1. Introduction

Cataract biometry is an essential component of cataract surgical planning. Accurate biometric measurement is critical for appropriate intraocular lens (IOL) selection and for achieving desired refractive outcomes. The Zeiss IOLMaster 700 uses swept-source optical coherence tomography (SS-OCT) technology to measure axial and anterior segment biometric parameters [1]. IOLMaster 700 measures anterior keratometry using telecentric keratometry from 18 reflected measurement points arranged on three concentric rings (1.5-, 2.5-, and 3.3 mm diameters) [2,3,4]. Posterior corneal curvature and central corneal thickness are measured from six meridional SS-OCT scans centered on the corneal apex (Jin et al., Wang et al., Savini et al.) [2,3,4]. These measurements are combined within the proprietary Total Keratometry (TK) algorithm, which applies a thick-lens optical model to estimate total keratometry from anterior keratometry, posterior keratometry, and pachymetry (Savini et al.) [4].

The Heidelberg Anterion Cataract App uses a 1300 nm SS-OCT system to acquire 65 radial B-scans and 256 A-scans per B-scan, allowing for a full three-dimensional reconstruction of the anterior and posterior corneal surfaces [5,6,7]. Anterion then directly derives anterior keratometry, posterior keratometry, pachymetry, and total keratometry from the tomographic reconstruction of the anterior segment [8]. The Anterion platform, consisting of the Cataract, Cornea, and Metrics Apps, received United States (US) Food and Drug Administration (FDA) clearance for clinical use in the US population in October 2023.

Few prior studies have compared biometry measurements obtained with the IOLMaster 700 and Anterion in European and Asian populations before US FDA approval [9,10]. However, several of these studies were limited by modest sample sizes, single-center designs, and recruitment from relatively ethnically homogeneous populations. In addition, some studies did not report the ethnic composition of their participants, which may limit interpretation given known differences in ocular anatomy across ethnic groups [11,12,13]. Although previous studies have compared concordance between the IOLMaster 700 and Anterion, their results may have limited generalizability to demographically diverse populations such as those in the US. Furthermore, prior studies have not evaluated the same parameters. For example, Oh et al. studied agreement between Anterion and IOLMaster 700 measurements for anterior and total keratometry, whereas Ang et al. and Panda et al. did not assess total keratometry. None of these studies compared posterior keratometry between the two devices [9,10,14].

This evidence gap is clinically relevant for US cataract surgeons in practices considering adoption of Anterion, transitioning from an existing biometer, or using the two devices across different clinical sites. Therefore, our goal was to examine concordance in cataract biometry, keratometry, and IOL calculation outputs between Anterion and IOLMaster 700 in a US cataract population to determine whether these devices could be considered interchangeable for practicing cataract surgeons.

## 2. Methods

### 2.1. Study Population

This was a retrospective cross-sectional study of patients presenting to the Wilmer Eye Institute, Johns Hopkins Medicine in Baltimore, MD and Bethesda, MD, from August 2024 to February 2025. The study included eyes evaluated for possible cataract surgery. Eyes worked up for cataract surgery that were found to have a clear crystalline lens remained eligible. We excluded eyes with any corneal abnormalities, including keratoconus, prior refractive surgery, Fuchs endothelial dystrophy, corneal edema, pterygium, or moderate to severe ocular surface disease. We also excluded eyes with a history of prior anterior segment surgery, such as cataract or glaucoma surgery. As a retrospective study, no a priori sample size calculation was performed; all eligible eyes imaged with both devices during the study period were included. The resulting sample is within the range of prior concordance studies of these devices and provided sufficient precision for estimation of the limits of agreement [14,15]. The study was approved by the Johns Hopkins University School of Medicine Institutional Review Board and adhered to the tenets of the Declaration of Helsinki.

### 2.2. Biometric Measurements

Eyes underwent biometry using SS-OCT (Heidelberg Anterion Cataract App, Heidelberg, Germany) and optical biometry (Zeiss IOLMaster 700, Oberkochen, Germany). Measurements were obtained in a real-world clinical setting by experienced ophthalmic technicians; for each patient, the same technician performed measurements on both devices. Parameters included axial length (AL), anterior chamber depth (ACD), lens thickness (LT), horizontal white-to-white distance (WTW), and pupil diameter (PD) all in millimeters; central corneal thickness (CCT) in micrometers; and anterior, posterior, and total keratometry (K) in diopters (D), each comprising K1, K2, average K, and difference in Ks (ΔK).

### 2.3. IOL Power Calculation

To determine the clinical relevance of any differences in biometric measurements from IOLMaster 700 versus Anterion in terms of non-toric IOL selection, we used measurements from each eye to perform IOL calculations using the Barrett Universal II “(https://calc.apacrs.org/barrett_universal2105/Default.aspx (Accessed from 1 March 2025–1 September 2025)”. For each eye and each machine, we entered relevant biometric parameters including AL, ACD, anterior Ks (K1 and K2), LT, and WTW into the formula and recorded the IOL power recommended assuming an A-constant of 119.1 (equivalent to an Alcon CC60WF monofocal IOL) and a distance refractive target of plano. Although anterior, posterior, and total keratometry were compared between devices, only anterior keratometry measurements were used as inputs for IOL calculations, consistent with conventional use of the Barrett Universal II formula. Each IOL power calculation generated an exact, continuous recommended lens power, which we defined as the unrounded spherical power. The rounded spherical power was defined as the Barrett Universal II-recommended spherical IOL power closest to plano after rounding to the nearest 0.50-D lens increment.

To determine clinical significance of any measurement differences in terms of toric IOL selection, calculations using each machine’s measurements were again performed using the Barrett Toric formula “https://ascrs.org/tools/barrett-toric-calculator (Accessed from 1 March 2025–1 September 2025)”, using a distance refractive target of plano, A-constant of 119.1, and surgically induced astigmatism of 0. Total keratometry measurements were not entered into the calculator. The online Barrett Toric proprietary formula uses anterior keratometry measurements to predict posterior corneal astigmatism to derive total corneal astigmatism [4,16].

### 2.4. Data Export and Statistical Analysis

Biometric measurements for eligible eyes were abstracted from each imaging platform and entered into the REDCap database [17,18]. All analyses were performed using Stata version 18.5 (StataCorp, College Station, TX, USA) for statistical analysis.

For each eye, paired measurements were compared for all biometric parameters. A complete-case analysis was performed for each parameter. Eyes contributed to a given comparison only if both devices returned a non-missing measurement for that parameter. No imputation was performed. Continuous data are presented as mean (standard deviation [SD]) or median (interquartile range) and categorical variables as counts (percentages). For each parameter, linear agreement was quantified using the Pearson correlation coefficient (ρ). Agreement was assessed using Lin’s concordance correlation coefficient (CCC), together with the bias-correction factor (Cb), which indexes the departure of the best-fit line from the line of identity [19]. The strength of linear agreement was interpreted as very strong: ρ ≥ 0.8; moderately strong: ρ ≥ 0.6; fair: ρ ≥ 0.3 and poor: ρ < 0.3 [20]. The strength of agreement indicated by the CCC was interpreted following the benchmark scheme of Partik et al. as excellent (>0.95), very good (0.90–0.95), fairly good (0.80–<0.90), middling/satisfactory (0.70–<0.80), mediocre (0.60–<0.70), poor (0.50–<0.60), or unacceptable (<0.50) [21]. Agreement was visualized with scatter plots against the line of identity (x = y) and Bland–Altman plots of the inter-device difference (Anterion - IOLMaster 700) against the mean of the two devices. We checked for presence of proportional bias and heteroscedasticity. Standard limits of agreement (LoA) were used when neither was present, and regression-based limits otherwise [22]. To account for within-patient clustering, 95% confidence intervals (CIs) for the mean difference and each LoA were estimated by cluster bootstrap (patient-level, 1000 replications) with percentile-based CIs. The mean inter-device difference was additionally estimated using an univariable generalized estimating equation (GEE) model (Gaussian family, identity link, independent working correlation, robust standard errors, clustered at the patient level).

For each eye, IOL power calculations derived from the two devices’ biometry were also compared for non-toric and toric lenses. For each calculation, the absolute inter-device difference (|Anterion - IOLMaster 700|) was computed per eye and categorized into clinically established agreement bands of <0.50 D, 0.50 to <1.00 D, 1.00 to <1.50 D, 1.50 to <2.00 D, and ≥2.00 D for IOL power, cylinder, and of <0.25 D, 0.25 to <0.50 D, 0.50 to <0.75 D, 0.75 to <1.00 D, and ≥1.00 D for refraction and residual astigmatism. The number and proportion of eyes within each band are tabulated and displayed as bar charts.

Two-sided *p*-values less than 0.05 and 95% CIs not overlapping the null were considered statistically significant.

## 3. Results

The demographic and clinical characteristics of the study population are summarized in Table 1 and Table 2. Data were obtained from 64 eyes of 40 patients. The median age was 70.00 years (IQR, 65.00–76.00), and 28 patients (70.00%) were female. The cohort was predominantly European American (n = 22, 55.00%), with smaller proportions of African American (n = 8, 20.00%), Asian (n = 6, 15.00%), and unknown race (n = 4, 10.00%); one patient (2.50%) identified as Hispanic. The median distance visual acuity was 0.30 logMAR, with an interquartile range corresponding to Snellen acuity of 20/25 to 20/40, and 49 eyes (76.56%) had Snellen visual acuity of 20/40 or better. Most eyes had some degree of cataract (n = 63, 98.44%), whereas a single eye (1.56%) had a clear crystalline lens. The most common cataract grades were 2+ nuclear sclerotic (n = 28, 43.75%), 2+ cortical (n = 16, 25.00%), and 3+ nuclear sclerotic (n = 14, 21.88%).

### 3.1. Biometry

**Figure 1:**
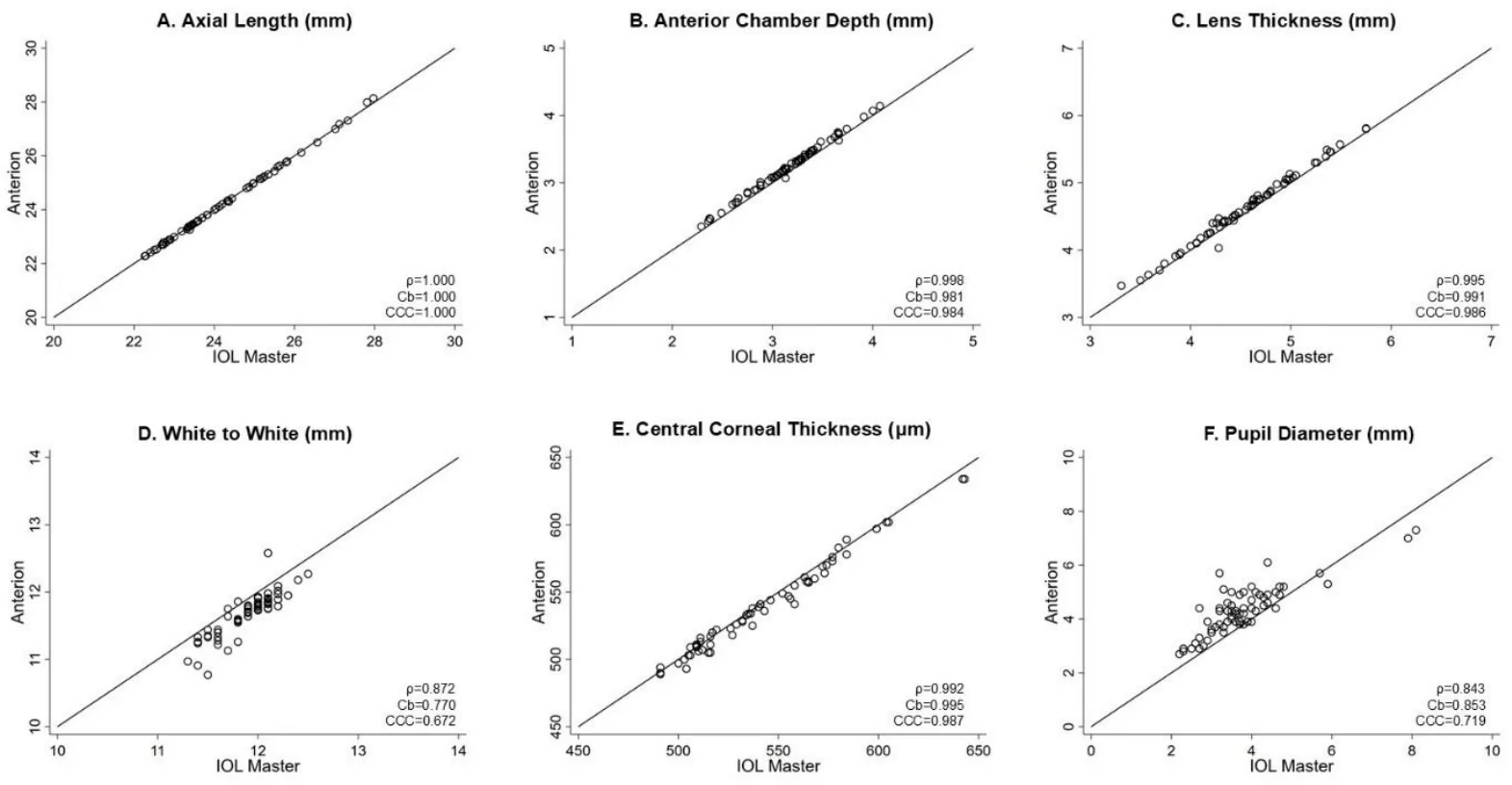
Scatter Plots Comparing Measurements Obtained by Anterion Versus IOLMaster 700. *Legend:* Abbreviations: mm = millimeters; μm = microns; ρ = Pearson correlation coefficient; CCC = Lin’s Concordance Coefficient Correlation; Cb = Bias correction factor. Each open circle represents an individual observation (eye) rather than an individual patient. The solid line represents the line of perfect agreement between the two devices (x = y).

The linear association and agreement between biometric measurements obtained with Anterion and IOLMaster 700 are presented in Figure 1. IOLMaster 700 and Anterion showed very strong linear relationships and excellent concordance for the principal biometric parameters, including AL (ρ = 1.000, CCC = 1.00), ACD (ρ = 0.998, CCC = 0.984), LT (ρ = 0.995, CCC = 0.986), and CCT (ρ = 0.992, CCC = 0.987). PD showed a very strong linear relationship and satisfactory concordance (ρ = 0.843, CCC = 0.719). The two devices also demonstrated a very strong linear relationship but mediocre concordance for WTW (ρ = 0.872, CCC = 0.672).

Table 3 summarizes the mean biometric values for each device and the mean inter-device difference, calculated as Anterion minus IOLMaster 700, estimated using generalized estimating equation models. AL was nearly identical between devices, with no statistically significant difference (β = −0.01 mm; 95% CI, −0.02 to 0.01; *p* = 0.46). Statistically significant inter-device differences were observed for all remaining parameters (*p* < 0.01 for each), although the absolute magnitudes were small. Compared with IOLMaster 700, Anterion yielded marginally higher values for ACD (mean difference = 0.07 mm; 95% CI, 0.06 to 0.08) and LT (mean difference = 0.07 mm; 95% CI, 0.06 to 0.08), and lower values for WTW (mean difference = −0.22 mm; 95% CI, −0.26 to −0.18) and CCT (mean difference = −3.19 μm; 95% CI, −4.48 to −1.90). The largest difference was observed for PD, with Anterion measuring 0.56 mm larger on average than IOLMaster 700 (95% CI, 0.38 to 0.73).

**Figure 2:**
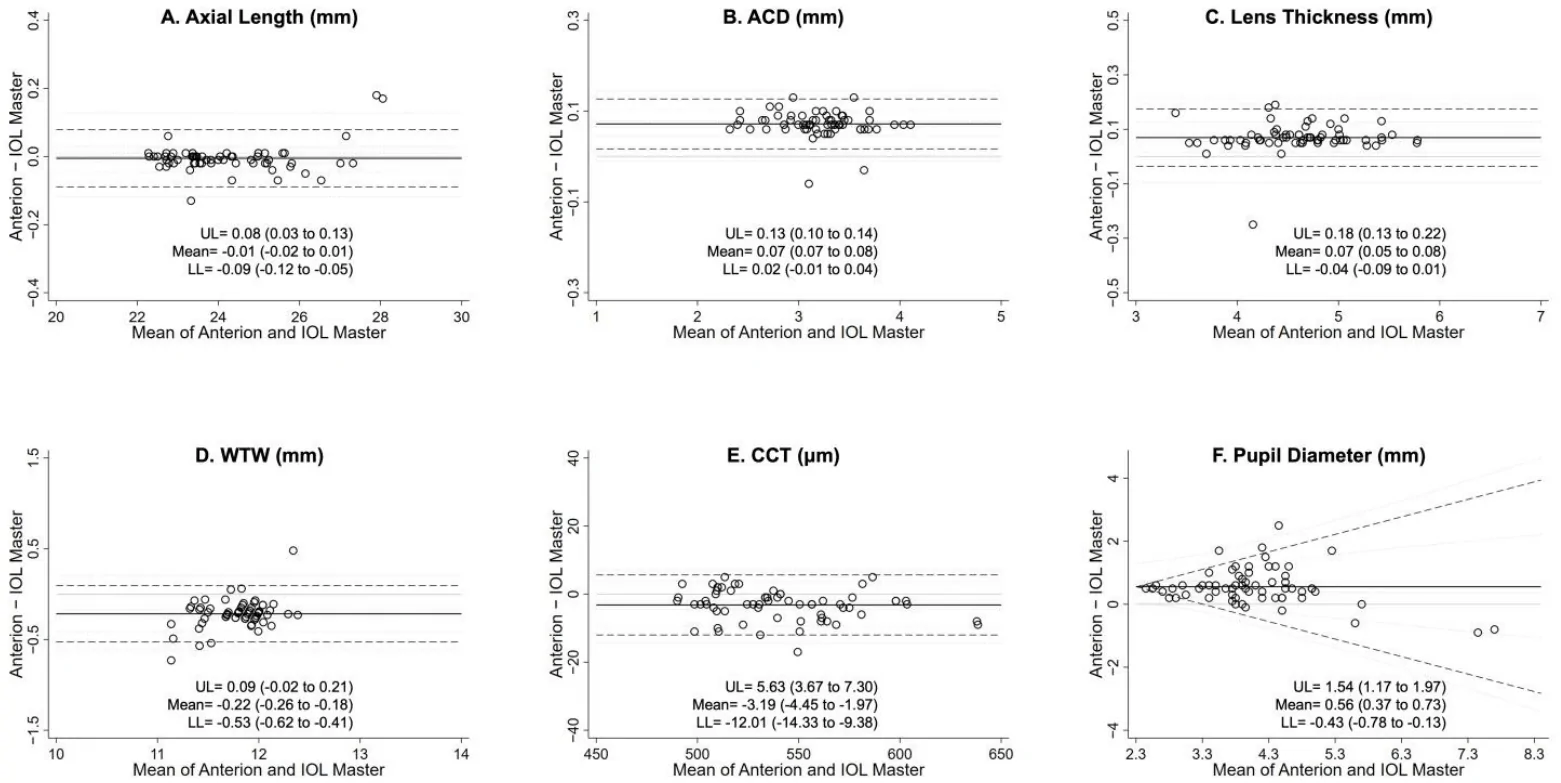
Bland–Altman Plots of Measurement Agreement Between Anterion and IOLMaster 700. *Legend:* Abbreviations: mm = millimeters; μm = microns; UL = upper limit, LL = lower limit. Bland–Altman plots show the difference between the two devices (*y*-axis) plotted against the average of the two devices (*x*-axis). Each open circle represents an individual observation (eye) rather than an individual patient. The solid black line indicates the mean difference (bias) between devices, and the dashed black lines indicate the upper and lower limits of agreement. Solid gray is the line of no difference. Small black dotted lines indicate the 95% percentile-based confidence intervals (CI) for the mean bias and limits of agreement, estimated using a clustered bootstrap to account for multiple observations per patient. 95% CI are stated in parenthesis.

Bland–Altman analysis (Figure 2) showed close agreement between the two devices, with narrow limits of agreement (LoA) for the core biometric parameters. For AL, the mean difference was negligible (−0.01 mm), and the 95% LoA extended from −0.09 mm (95% CI, −0.12 to −0.05) to 0.08 mm (95% CI, 0.03 to 0.13). ACD and LT also showed tight limits, spanning 0.02 to 0.13 mm and −0.04 to 0.18 mm, respectively. Wider limits were observed for WTW (−0.53 to 0.09 mm) and CCT (−12.01 to 5.63 μm). The widest limits were observed for PD, ranging from −0.43 mm (95% CI, −0.78 to −0.13) to 1.54 mm (95% CI, 1.17 to 1.97). No parameter demonstrated statistically significant proportional bias (all *p* ≥ 0.06), indicating that the inter-device difference did not vary systematically with measurement magnitude. However, statistically significant heteroscedasticity was detected for PD (*p* < 0.01) only, indicating that the spread of inter-device differences widened with increasing mean pupil size.

### 3.2. Keratometry Concordance

**Figure 3:**
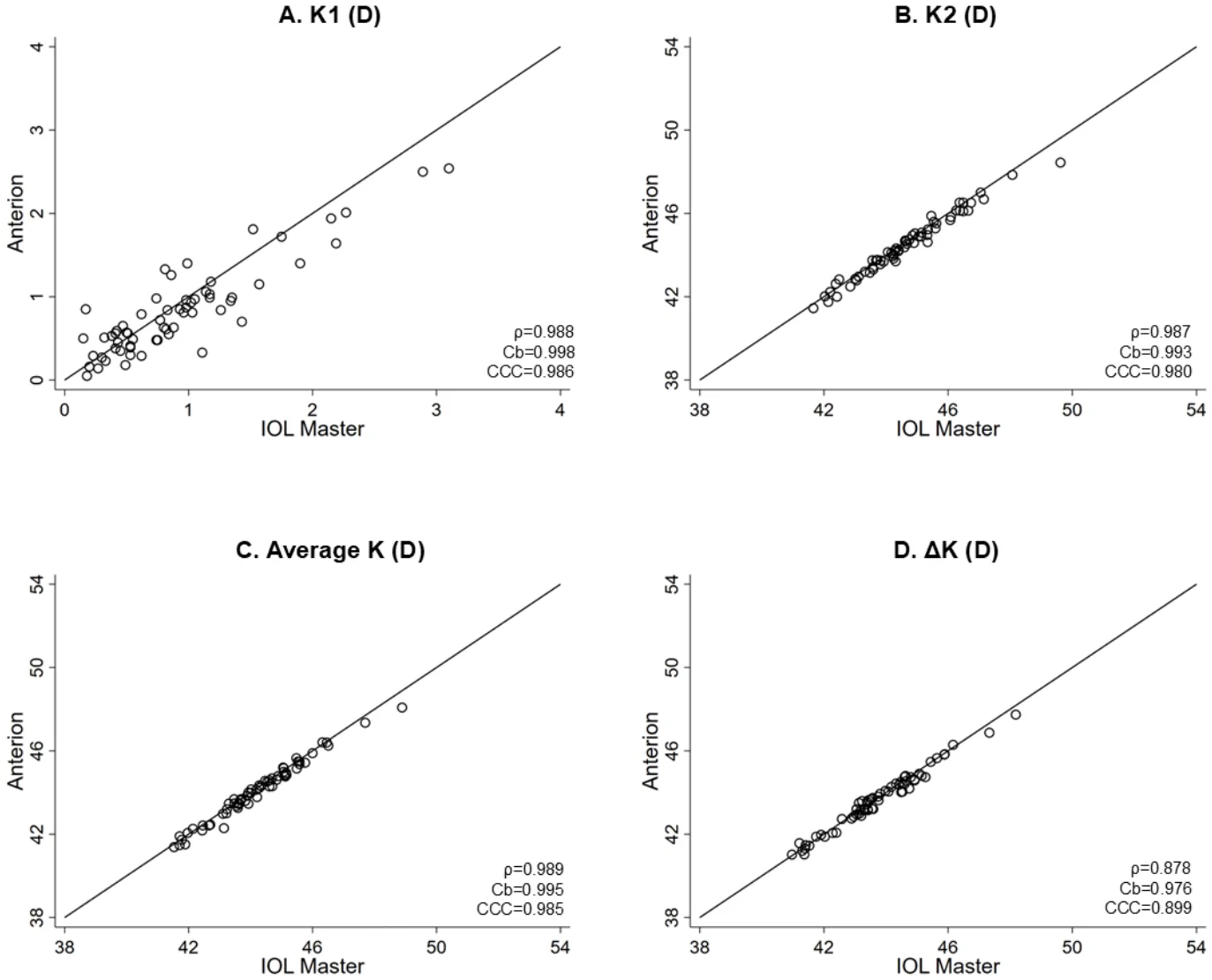
Scatter Plots Comparing Anterior Keratometry Obtained by Anterion Versus IOLMaster 700. *Legend:* Abbreviations: D = Diopters; ρ = Pearson correlation coefficient; CCC = Lin’s Concordance Coefficient Correlation; Cb = Bias correction factor; Δ = Difference. Each open circle represents an individual observation (eye) rather than an individual patient. The solid line represents the line of perfect agreement between the two devices (x = y).

**Figure 4:**
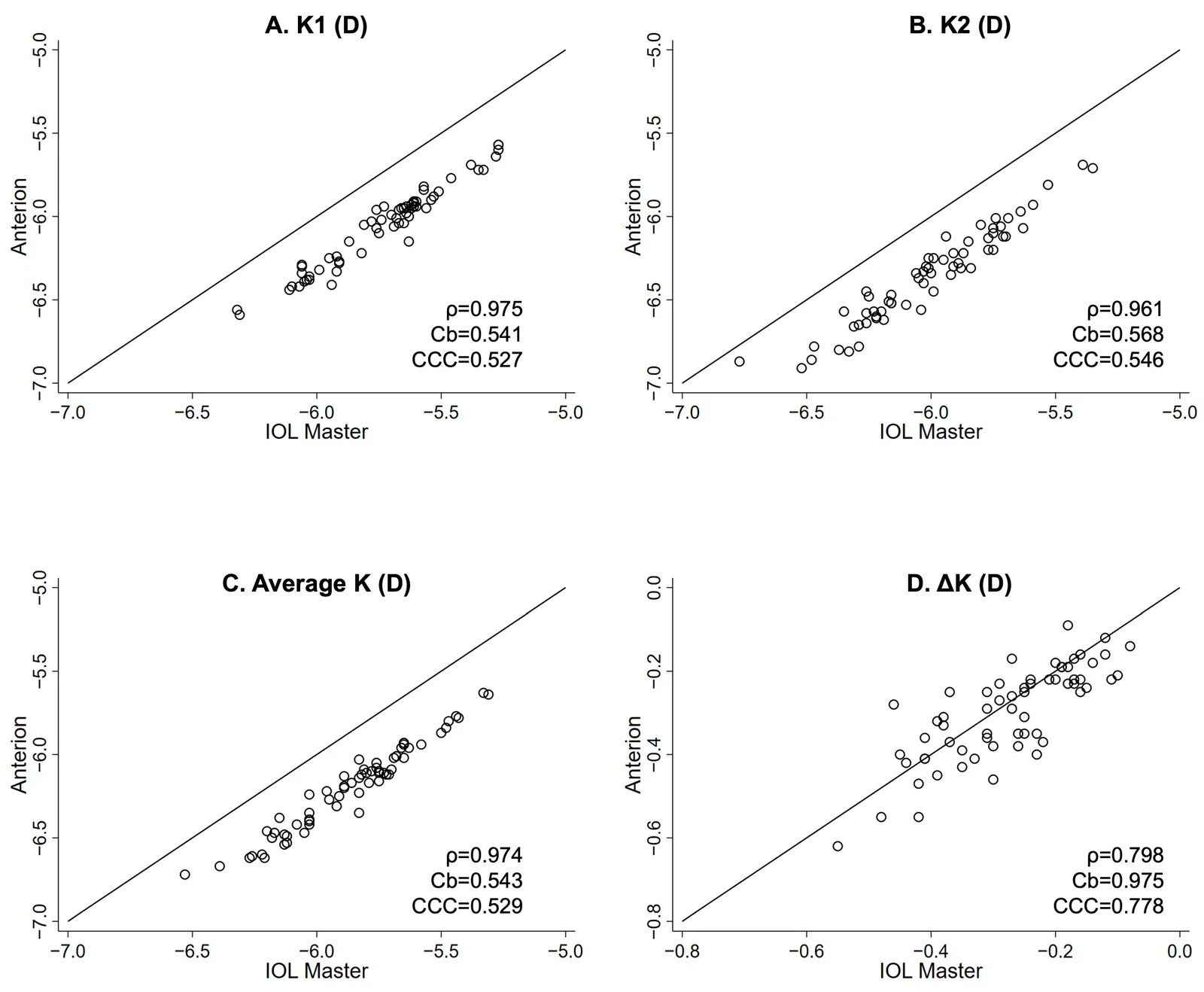
Scatter Plots Comparing Posterior Keratometry Obtained by Anterion Versus IOLMaster 700. *Legend:* Abbreviations: D = Diopters; ρ = Pearson correlation coefficient; CCC = Lin’s Concordance Coefficient Correlation; Cb = Bias correction factor; Δ = Difference. Each open circle represents an individual observation (eye) rather than an individual patient. The solid line represents the line of perfect agreement between the two devices (x = y).

**Figure 5:**
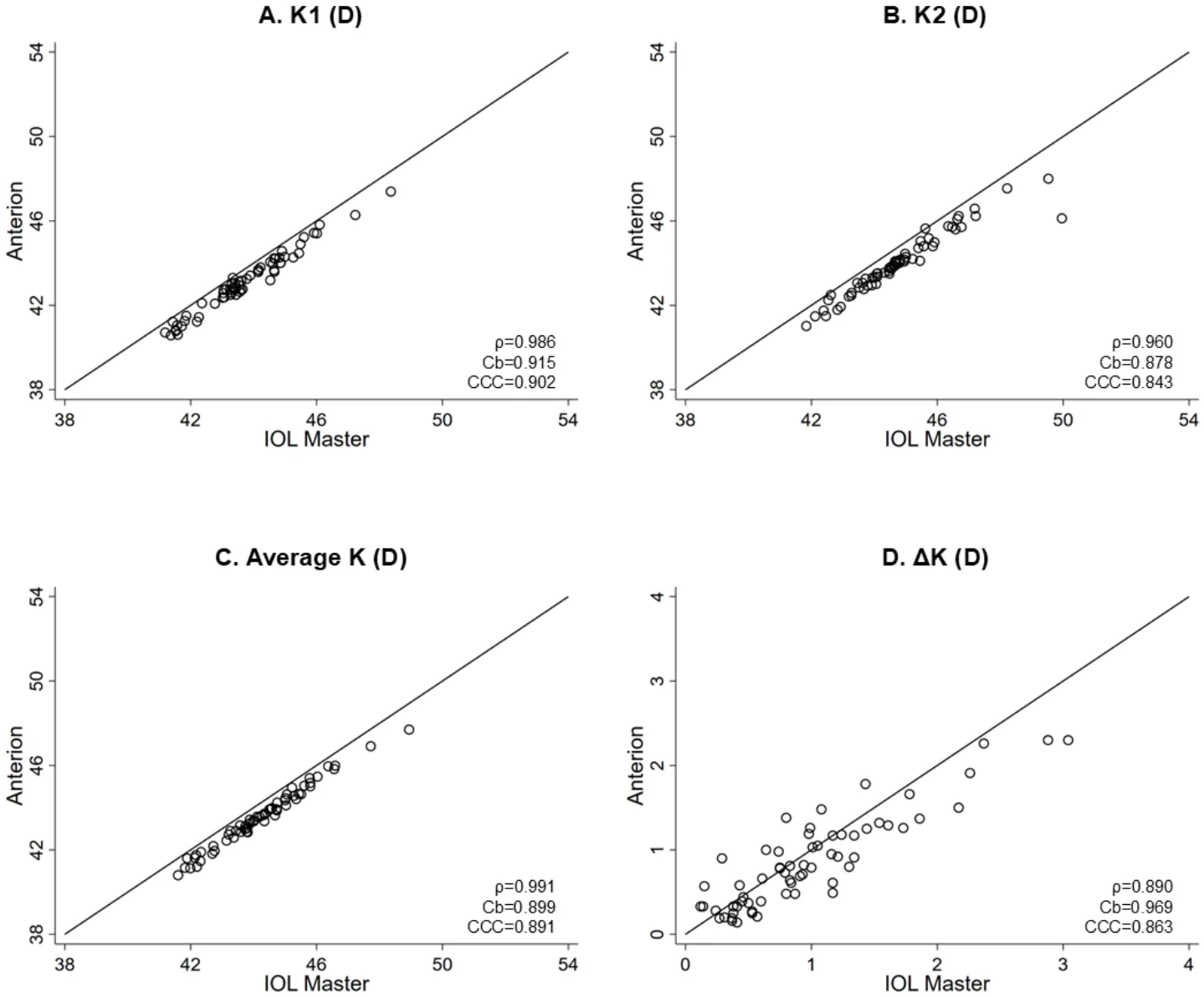
Scatter Plots Comparing Total Keratometry Obtained by Anterion Versus IOLMaster 700. *Legend:* Abbreviations: D = Diopters; ρ = Pearson correlation coefficient; CCC = Lin’s Concordance Coefficient Correlation; Cb = Bias correction factor; Δ = Difference. Each open circle represents an individual observation (eye) rather than an individual patient. The solid line represents the line of perfect agreement between the two devices (x = y).

The linear association and agreement between the two devices for anterior, posterior, and total keratometry are presented in Figure 3, Figure 4, and Figure 5, respectively. For anterior keratometry, IOLMaster 700 and Anterion showed very strong linear relationships and excellent concordance for K1 (ρ = 0.988, CCC = 0.986), K2 (ρ = 0.987, CCC = 0.980), and average K (ρ = 0.989, CCC = 0.985) with very strong correlation and very good concordance for ΔK (ρ = 0.878, CCC = 0.899). Total keratometry showed a similar pattern, with very strong linear relationships across all parameters and concordance ranging from fairly good to very good for K1 (ρ = 0.986, CCC = 0.902), K2 (ρ = 0.960, CCC = 0.843), average K (ρ = 0.991, CCC = 0.891), and ΔK (ρ = 0.890, CCC = 0.863). In contrast, posterior keratometry showed very strong linear relationships but poor concordance for K1 (ρ = 0.975, CCC = 0.527), K2 (ρ = 0.961, CCC = 0.546) and average K (ρ = 0.974, CCC = 0.529). Concordance for posterior ΔK was satisfactory (ρ = 0.798, CCC = 0.778).

Mean keratometric values for each device and corresponding inter-device differences are presented in Table 4. Statistically significant differences were observed between devices for all corneal curvature parameters (*p* ≤ 0.02). For anterior keratometry, the differences were small, with Anterion measuring marginally flatter than IOLMaster 700 for K1 (mean difference = −0.08 D; 95% CI, −0.14 to −0.02), K2 (mean difference = −0.16 D; 95% CI, −0.23 to −0.10) and average K (mean difference = −0.13 D; 95% CI, −0.19 to −0.08), with a similarly small difference in corneal astigmatism magnitude (ΔK mean difference = −0.10 D; 95% CI, −0.16 to −0.03). The largest discrepancies were observed for posterior keratometry, for which Anterion consistently yielded more negative, or steeper, values than IOLMaster 700 for K1 (mean difference = −0.32 D; 95% CI, −0.34 to −0.31), K2 (mean difference = −0.35 D; 95% CI, −0.37 to −0.33) and average K (mean difference = −0.34 D; 95% CI, −0.35 to −0.32). For total keratometry, Anterion measured flatter values than IOLMaster 700, with differences of −0.62 D (95% CI, −0.69 to −0.55) for K1, and −0.79 D (95% CI, −0.90 to −0.68) for K2, −0.68 D (95% CI, −0.73 to −0.62) for average K along with a small difference in astigmatism magnitude (ΔK mean difference = −0.12 D; 95% CI, −0.19 to −0.04).

**Figure 6:**
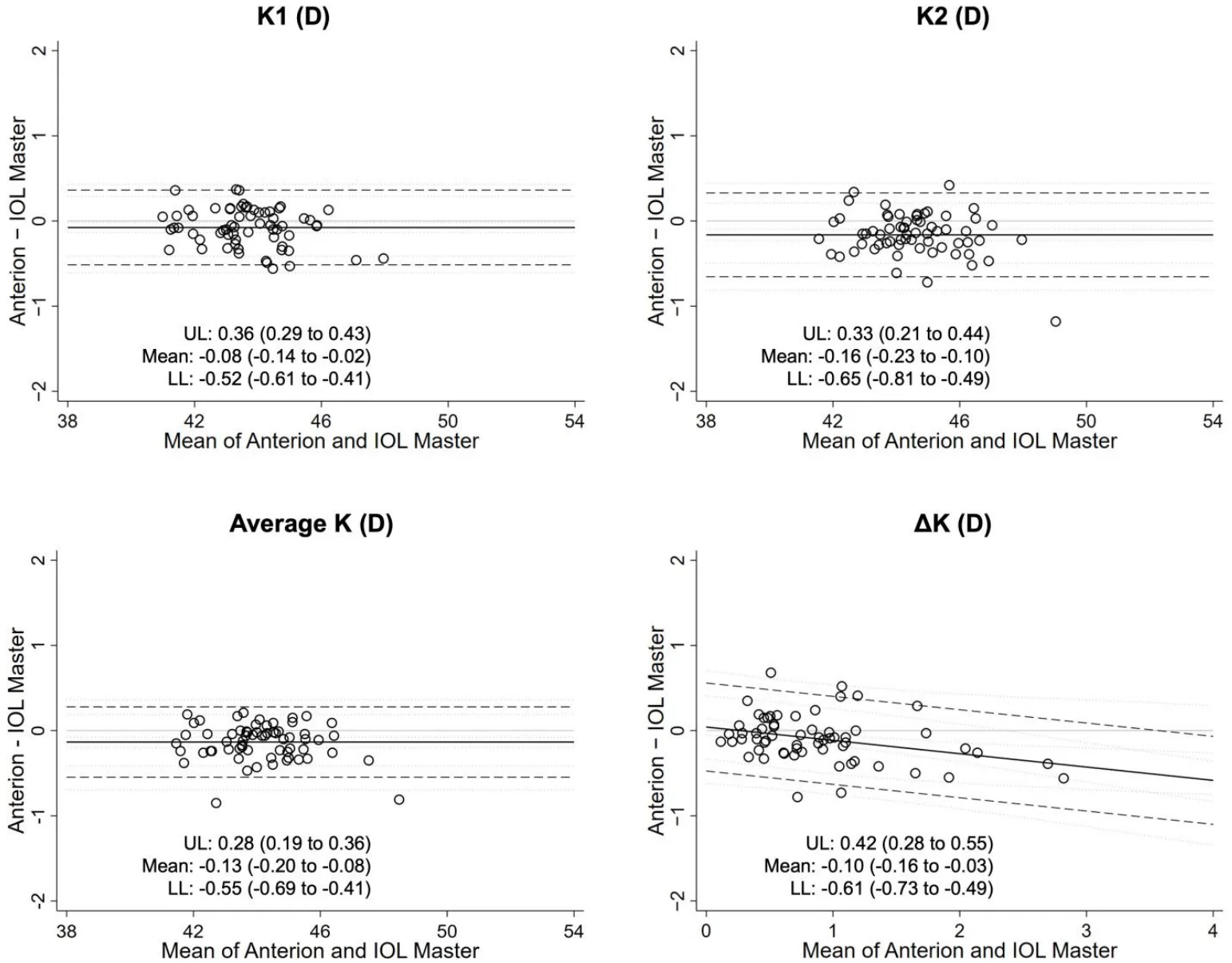
Bland–Altman Plots of Anterior Keratometry Agreement Between Anterion and IOLMaster 700. *Legend:* Abbreviations: D = diopters, UL = upper limit, LL = lower limit. Bland–Altman plots show the difference between the two devices (*y*-axis) plotted against the average of the two devices (*x*-axis). Each open circle represents an individual observation (eye) rather than an individual patient. The solid black line indicates the mean difference (bias) between devices, and the dashed black lines indicate the upper and lower limits of agreement. Solid gray is the line of no difference. Small black dotted lines indicate the 95% percentile-based confidence intervals (CI) for the mean bias and limits of agreement, estimated using a clustered bootstrap to account for multiple observations per patient. 95% CI are stated in parenthesis.

**Figure 7:**
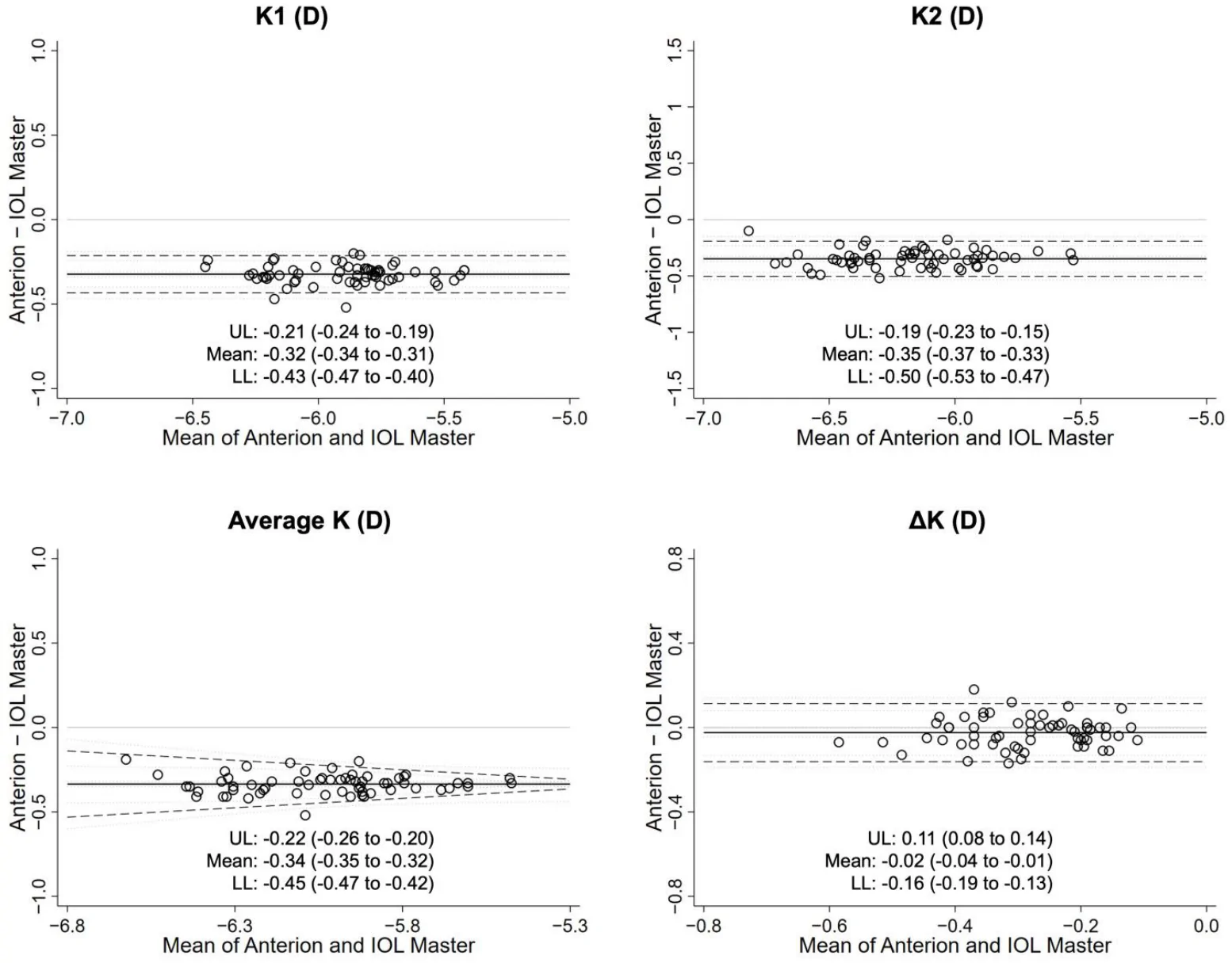
Bland–Altman Plots of Posterior Keratometry Agreement Between Anterion and IOLMaster 700. *Legend:* Abbreviations: D = diopters, UL = upper limit, LL = lower limit. Bland–Altman plots show the difference between the two devices (*y*-axis) plotted against the average of the two devices (*x*-axis). Each open circle represents an individual observation (eye) rather than an individual patient. The solid black line indicates the mean difference (bias) between devices, and the dashed black lines indicate the upper and lower limits of agreement. Solid gray is the line of no difference. Small black dotted lines indicate the 95% percentile-based confidence intervals (CI) for the mean bias and limits of agreement, estimated using a clustered bootstrap to account for multiple observations per patient. 95% CI are stated in parenthesis.

**Figure 8:**
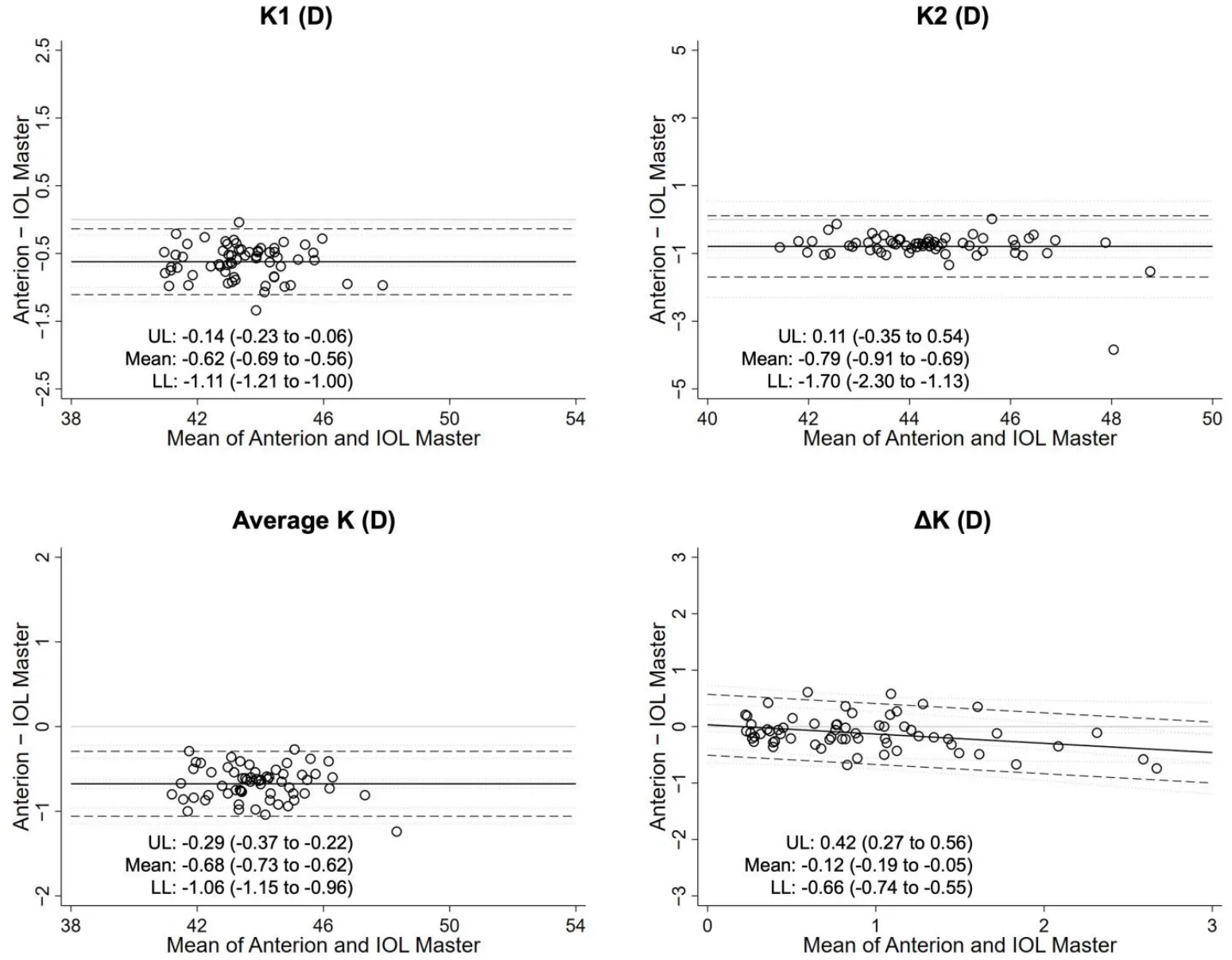
Bland–Altman Plots of Total Keratometry Agreement Between Anterion and IOLMaster 700. *Legend:* Abbreviations: D = diopters, UL = upper limit, LL = lower limit. Bland–Altman plots show the difference between the two devices (*y*-axis) plotted against the average of the two devices (*x*-axis). Each open circle represents an individual observation (eye) rather than an individual patient. The solid black line indicates the mean difference (bias) between devices, and the dashed black lines indicate the upper and lower limits of agreement. Solid gray is the line of no difference. Small black dotted lines indicate the 95% percentile-based confidence intervals (CI) for the mean bias and limits of agreement, estimated using a clustered bootstrap to account for multiple observations per patient. 95% CI are stated in parenthesis.

Bland–Altman analysis for anterior, posterior, and total keratometry is presented in Figure 6, Figure 7 and Figure 8. For anterior keratometry, the LoA were narrow across all keratometric indices, with upper limits ranging from 0.28 to 0.42 D and lower limits ranging from −0.52 to −0.65 D. Posterior keratometry showed the narrowest limits in absolute width. For the posterior curvature parameters (average K, K1, and K2), both bounds were consistently offset below zero, reflecting the systematic inter-device difference noted above. In contrast, the limits for posterior ΔK straddled zero (mean difference −0.02 D, 95% LoA −0.16 to 0.11 D), consistent with the inter-device offset affecting both posterior meridians similarly. Total keratometry showed the widest limits, particularly for K2, with limits ranging from −1.70 D (95% CI, −2.30 to −1.13) to 0.11 D (95% CI, −0.35 to 0.54). Statistically significant proportional bias was detected for anterior ΔK (*p* < 0.01) and total ΔK (*p* = 0.01), indicating that the inter-device difference in astigmatism magnitude varied with the mean value. Statistically significant heteroscedasticity was detected only for posterior average K (*p* = 0.01), indicating non-uniform scatter of differences across the measurement range.

### 3.3. Non-Toric and Toric IOL Calculations

**Figure 9:**
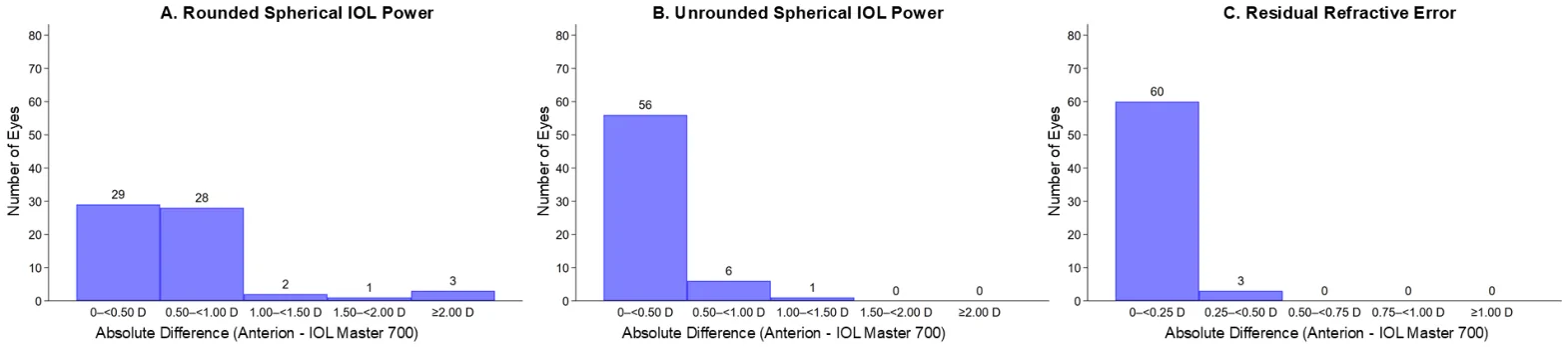
Comparison of Non-Toric IOL Calculations Between Anterion and IOLMaster 700. *Legend:* (**A**) represents the absolute difference in rounded spherical power lens selection between Anterion and IOLMaster 700. (**B**) represents the absolute difference in unrounded spherical power lens selection between Anterion and IOLMaster 700. (**C**) represents the absolute difference in residual refractive error based on Barret Universal II IOL calculations.

Agreement between the two devices for non-toric IOL calculations is presented in Figure 9. For rounded spherical lens power, the absolute inter-device difference was within 0.50 D in 29 of 63 eyes (46.03%) and within the 0.50–<1.00 D band in 28 eyes (44.44%). Six eyes (9.52%) showed differences of 1.00 D or greater, including 3 eyes (4.76%) that differed by 2.00 D or more. When unrounded calculated spherical power was compared, absolute differences were within 0.50 D in 56 eyes (88.88%) and within 1.00 D in 62 eyes (98.41%). Only 1 eye (1.58%) exceeded 1.00 D, and no eye differed by 1.50 D or more. The smallest absolute differences were observed for predicted residual refractive error, which was within 0.25 D in 60 of 63 eyes (95.24%) and within 0.50 D in all eyes.

**Figure 10:**
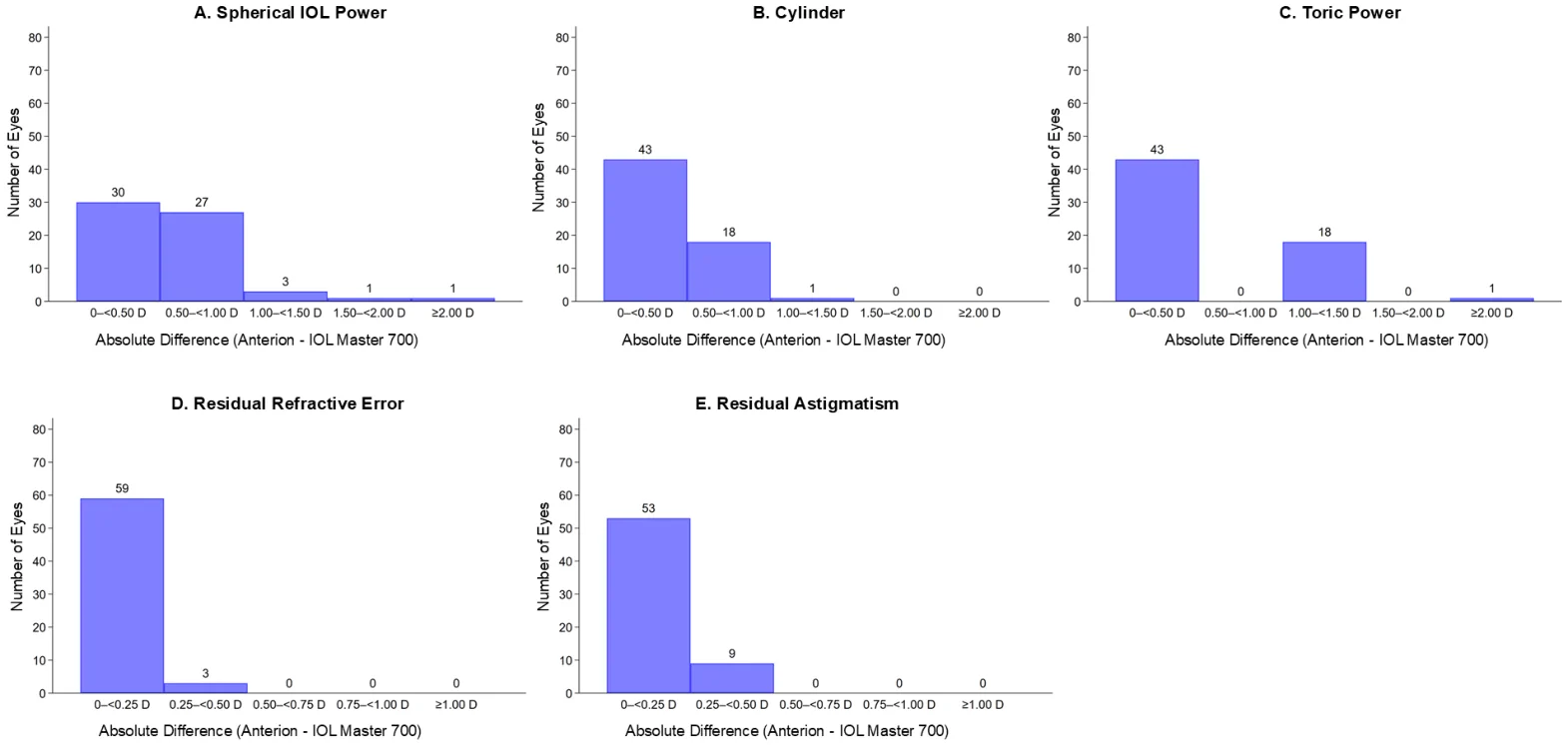
Comparison of Toric IOL Calculations Between Anterion and IOLMaster 700. *Legend:* (**A**) represents the absolute difference in spherical power lens selection between Anterion and IOLMaster 700. (**B**) represents the absolute difference in cylinder selection between Anterion and IOLMaster 700. (**C**) represents the absolute difference in Toric Power selection between Anterion and IOLMaster 700. (**D**) represents the absolute difference in residual refractive error based on the Barret True-K Calculations using Anterion versus IOLMaster 700. (**E**) represents the absolute difference in residual astigmatism between Anterion and IOLMaster 700.

Agreement between the two devices for toric IOL calculations is presented in Figure 10. For spherical power selection, the absolute inter-device difference was within 0.50 D in 30 of 62 eyes (48.39%) and within the 0.50–<1.00 D band in 27 eyes (43.55%). Five eyes (8.06%) differed by 1.00 D or more, including 1 eye (1.61%) that differed by 2.00 D or more. For cylinder selection, the difference was within 0.50 D in 43 eyes (69.35%) and within 1.00 D in 61 eyes (98.39%), with only 1 eye (1.61%) differing by 1.00 D or more. For toric IOL power, 43 eyes (69.35%) were within 0.50 D, and 18 eyes (29.03%) fell within the 1.00–<1.50 D band, with 1 eye (1.61%) differing by 2.00 D or more. The smallest differences were again observed for predicted residual refractive error, which was within 0.25 D in 59 of 62 eyes (95.16%) and within 0.50 D in all eyes. For predicted residual astigmatism, the absolute difference was within 0.25 D in 53 eyes (85.48%) and within 0.50 D in all eyes.

## 4. Discussion

Compared with IOLMaster 700, the Anterion Cataract App showed close agreement for the biometric and anterior keratometric measurements most relevant to routine IOL power calculation, including AL and anterior K1, K2, and average K. AL was nearly identical between devices, and anterior keratometry demonstrated near-perfect concordance, suggesting that the two devices provide highly similar inputs for standard cataract planning. Although Anterion measured statistically significantly greater ACD, LT, and PD values and slightly lower WTW and CCT values than IOLMaster 700, these differences were small in magnitude, and their clinical significance is likely limited for routine IOL power selection. Importantly, these differences did not translate into large predicted refractive differences for most eyes: predicted residual refractive error differed by <0.25 D in more than 95.24% of eyes for both non-toric and toric calculations, and predicted residual astigmatism differed by <0.25 D in 85.48% of eyes. These findings suggest that Anterion and IOLMaster 700 provide comparable clinically relevant IOL calculation outputs when anterior keratometry based calculations are performed for most cataract surgery candidates. However, since Anterion systematically measured steeper posterior keratometry and flatter total keratometry than IOLMaster 700, they cannot be considered interchangeable for these parameters.

### 4.1. Non-Toric IOL Calculations

The discrepancy between rounded and unrounded spherical IOL power agreement may have practical implications for cataract surgery planning. Modern IOL formulas generate continuous power estimates and predicted postoperative refractions, whereas surgeons must select from commercially available IOLs in discrete increments, most commonly 0.50 D. Small inter-device differences in formula-derived power can therefore be amplified after rounding when two calculations fall on opposite sides of a rounding threshold. This threshold effect may explain why rounded non-toric spherical IOL power selection differed between devices in a substantial proportion of eyes in our cohort, whereas the unrounded formula-derived powers showed much closer agreement. The clinical significance of a rounded difference depends on the intended refractive target. Selection of the higher-powered IOL generally shifts the predicted postoperative refraction toward myopia, whereas the lower-powered IOL shifts it toward hyperopia. Therefore, adjacent IOL power selections may leave slight myopia versus slight hyperopia in an eye targeted for emmetropia, or greater versus lesser residual myopia in an eye targeted for near vision.

Prior studies comparing IOL calculations between Anterion and IOLMaster 700 have reported mixed findings. Using the Barrett Universal II formula, Dong et al. found that Anterion produced significantly higher spherical IOL powers than IOLMaster 700, Tañá-Sanz et al. found no significant difference between devices, and Shetty et al. found that IOLMaster 700 produced higher spherical IOL powers than Anterion [23,24,25,26]. In our cohort, unrounded non-toric spherical power differed between devices by <0.50 D in 88.88% of eyes, whereas rounded non-toric spherical power selection differed by <0.50 D in 46.03% and by 0.50–1.00 D in 44.44%. This distinction is clinically important, as IOL formula performance is typically evaluated using refractive prediction error and the proportion of eyes within clinically meaningful refractive thresholds rather than agreement in rounded IOL selection alone. Consistent with this framework, Pfaeffli et al. [15] found no significant differences in predicted residual refractive error between Anterion and IOLMaster 700. In our cohort, predicted residual refractive error differed by <0.25 D in 95.24% of eyes, suggesting that the underlying non-toric IOL calculations were highly similar for most eyes despite apparent differences in rounded IOL recommendations.

### 4.2. Toric IOL Calculations

Toric IOL calculations demonstrated that measurable inter-device differences in keratometry did not usually produce clinically meaningful differences in predicted refractive outcomes. Toric IOL planning depends on accurate measurement of corneal astigmatism magnitude and axis, including the contribution of posterior corneal astigmatism, which can alter estimates of total corneal astigmatism and influence toric IOL power selection [1,5,6]. In the present study, toric calculations were performed using the Barrett Toric Calculator with anterior keratometry measurements from each device. For eyes without previous corneal refractive surgery, the Barrett Toric Calculator estimates the effect of posterior corneal astigmatism using a mathematical model derived from anterior corneal measurements rather than directly incorporating measured total keratometry [4,16,27,28]. Using an anterior keratometry-based approach, predicted toric residual refractive error differed by <0.25 D in 95.16% of eyes, and predicted residual astigmatism differed by <0.25 D in 85.48%. Differences in discrete toric lens selection were more common: cylinder power selection differed by 0.50–1.00 D in 29.03% of eyes, and toric IOL power selection differed by 1.00–1.50 D in 29.03%. These findings could reflect threshold effects in toric lens selection, where small differences in measured astigmatism can move an eye across a manufacturer’s cylinder-power cutoff without substantially changing the predicted postoperative refractive result, or they could represent systematic differences in toric power recommendations across the two devices. This distinction is clinically important because residual astigmatism can reduce uncorrected visual acuity and patient satisfaction after cataract surgery, and residual cylinder also contributes to spherical equivalent refractive error because spherical equivalent incorporates one-half of the cylindrical error [9,10]. Overall, the predicted toric refractive outcomes for Anterion and IOLMaster 700 were highly similar for most eyes in this cohort. More studies are needed to replicate these findings and evaluate potential causes and clinical implications of differences in discrete toric lens power recommendations.

### 4.3. Posterior and Total Keratometry

Posterior and total keratometry were the principal areas of disagreement between devices, likely because these measurements are more dependent on device-specific acquisition and reconstruction than conventional anterior keratometry. IOLMaster 700 combines telecentric anterior keratometry with posterior corneal and pachymetric information obtained from six meridional SS-OCT scans, whereas Anterion reconstructs the anterior and posterior corneal surfaces from 65 radial B-scans comprising 16,640 data points across an 8 mm zone [2,4,7]. Previous studies have shown that Anterion and IOLMaster 700 have a mean posterior curvature difference of −0.38 D (K1) and −0.36 D (K2), which is similar to our results [29]. Differences in device acquisition of posterior curvature can contribute to the statistically significant differences we found in total keratometry measurements between the two devices. Oh et al. found that Anterion measured statistically smaller values for total K1 (mean difference −0.708) and total K2 (mean difference −0.525) [14]. McLintock et al. found similar results, demonstrating a mean difference of −0.82 for total K1, and −0.65 for total K2 [29]. Differences in scan geometry, sampled corneal area, segmentation, and proprietary processing could produce systematic differences in posterior keratometry. Since posterior keratometry is incorporated into the calculation of total keratometry, differences in posterior keratometry measurements can subsequently propagate into creating differences in total keratometry measurements. Therefore, the posterior and total keratometry measurements generated by the two devices should not be considered technically equivalent.

The observed posterior and total keratometry differences may become clinically relevant when these measurements are incorporated directly into IOL planning. This may be particularly important in eyes with previous LASIK or PRK, where corneal ablation alters the normal relationship between anterior and posterior corneal curvature and may reduce the accuracy of conventional keratometric assumptions [27,28,30]. Incorporation of total keratometry into the Barrett True-K TK formula has demonstrated favorable refractive prediction accuracy after laser refractive surgery [30,31,32]. This scenario was not evaluated because eyes with previous refractive surgery were excluded and all IOL calculations used anterior keratometry inputs. Future studies should determine whether the observed interdevice differences in posterior and total keratometry alter total keratometry-based spherical or toric IOL recommendations and postoperative refractive outcomes in eyes with a history of refractive surgery.

### 4.4. Agreement in Additional Biometry

Anterion consistently measured a slightly deeper ACD than IOLMaster (mean difference 0.07 mm, *p* < 0.001) with an excellent concordance between the two devices (CCC = 0.984). A possible explanation for this difference is that Anterion does not directly calculate ACD, but measures ACD by adding aqueous depth (AQD) to CCT. Previous studies have found similar results, showing Anterion to measure deeper ACD values when compared to IOLMaster 700 [9,10,14,33]. Anterion obtained 0.56 mm larger pupillary diameter and the two devices had satisfactory agreement (CCC = 0.719). Potential reasons for the differences in pupil diameter across the two devices include differences in patient positioning, different requirements for attempted accommodation when looking at each machine’s visual target, or different levels of ambient light emitted by each device’s screen. The clinical implications of any differences in pupil measurements need to be explored further. LT consistently measured 0.07 mm thicker using Anterion vs. IOLMaster. This minor difference is unlikely to be clinically significant for cataract surgical planning. Since LT measurements were highly correlated across the two devices, measurement differences are likely inherent to the imaging technologies used. WTW was consistently measured as smaller using Anterion vs. IOLMaster (mean difference −0.22 mm, 95% CI −0.26 to −0.18). The concordance between two machines’ measurements for horizontal WTW was mediocre (CCC = 0.672) and Anterion measured a smaller WTW than IOLMaster in all but one eye, indicating these differences are likely due to the imaging technology itself rather than measurement imprecision. The difference in horizontal WTW measurement across the two devices is unlikely to be clinically significant for most IOL calculation formulas, where WTW is a minor covariate without major impact on determination of IOL power. However, the consistent difference in WTW measurements across the two devices may be relevant for other scenarios such as anterior chamber IOL (ACIOL) implantation, secondary IOL implantation, corneal transplantation, and other anterior segment surgeries.

### 4.5. Strengths and Weaknesses

A major strength of this study is its comprehensive evaluation of the Anterion Cataract App and IOLMaster 700 across multiple levels of cataract biometry and surgical planning. Rather than limiting the comparison to standard biometry, anterior keratometry alone, or spherical IOL calculations, we evaluated agreement across biometric, anterior keratometric, posterior keratometric, and total keratometric measurements. This allowed us to distinguish areas of strong inter-device agreement from areas with systematic differences, particularly posterior and total keratometry. We also extended the analysis beyond measurement agreement by evaluating whether these differences affected clinically relevant outputs for IOL calculations. By including both non-toric and toric IOL calculations, as well as predicted residual refractive error and predicted residual astigmatism, this study provides a practical assessment of whether device-level measurement differences are likely to influence cataract surgery planning. This broader approach addresses an important gap in the literature, as prior studies have often evaluated only selected biometric parameters, anterior keratometry, or non-toric IOL calculations rather than linking anterior, posterior, and total corneal measurements to both spherical and astigmatic refractive planning outcomes.

This study also has limitations. The sample size was modest, limiting subgroup analyses by axial length, cataract density, astigmatism magnitude, and other ocular characteristics. We evaluated predicted rather than actual postoperative refractive outcomes, so future outcomes studies should assess refractive prediction error and residual astigmatism after cataract surgery using IOLs selected from each device. Different technicians obtained scans for different patients, which reflects real-world practice but may have introduced technician-level variability. Repeated scans were not obtained for all eyes, preventing assessment of within-device repeatability. Finally, our IOL calculations used only the Barrett Universal II and Barrett Toric formulas, whereas some prior studies evaluated multiple formulas; agreement between devices may differ with other formulas, lens platforms, surgically induced astigmatism assumptions, or surgeon-specific constants.

## 5. Conclusions

The Anterion Cataract App and IOLMaster 700 showed excellent agreement for AL, ACD, LT, and anterior keratometry, which are key inputs for cataract surgery planning. Anterion tended to measure steeper posterior keratometry and flatter total keratometry than IOLMaster 700. IOL calculation results were highly similar between the two devices with unrounded spherical lens power recommendation differing by <0.50 D in 88.88% of eyes, although potential differences in toric IOL cylindrical power calculations warrant further exploration.

## Figures and Tables

**Table 1:** Demographic and Clinical Characteristics of Patient Population.

Characteristics	Values
N, eyes	64
N, patients	40
Age, median (IQR)	70.00 (65.00, 76.00)
**Laterality, N (%)**	
Right eye	30 (46.88)
Left eye	34 (53.12)
**Gender, N (%)**	
Female	28 (70.00)
Male	12 (30.00)
**Race, N (%)**	
European American	22 (55.00)
African American	8 (20.00)
Asian	6 (15.00)
Unknown	4 (10.00)
Hispanic ethnicity	1 (2.50)
Intraocular pressure, mean (SD)	14.31 (3.24)
**Snellen visual acuity, N (%)**	
20/20 or lower	11 (17.19)
>20/20 to ≤20/40	38 (59.38)
>20/40 to ≤20/80	10 (15.62)
20/80 or higher	4 (6.25)
Unknown	1 (1.56)
**LogMAR visual acuity, median (IQR)**	0.30 (0.10, 0.30)
**Lens status**	
Clear crystalline lens	1 (1.56)
Cataract	63 (98.44)

Abbreviations: N = Number; IQR = Interquartile range; SD = Standard deviation.

**Table 2:** Proportion of Eyes by Cataract Type and Grade.

Cataract Grade	Nuclear Sclerotic Cataract, N (%)	Cortical Cataract, N (%)	Posterior Subcapsular Cataract, N (%)
None	2 (3.12)	17 (26.56)	49 (76.56)
Trace	2 (3.12)	12 (18.75)	0
Trace to 1+	0	0	0
1+	7 (10.94)	11 (17.19)	4 (6.25)
1 to 2+	3 (4.69)	0	2 (3.12)
2+	28 (43.75)	16 (25.00)	3 (4.69)
2 to 3+	4 (6.25)	1 (1.56)	1 (1.56)
3+	14 (21.88)	4 (6.25)	2 (3.12)
3 to 4+	1 (1.56)	0	0
Unknown	3 (4.69)	3 (4.69)	3 (4.69)

Abbreviations: Denominator = 64 eyes; N = Number.

**Table 3:** Association of Biometric Measurements with Anterion and IOLMaster 700.

Measurement Type	N (Eyes)	Anterion, Mean (SD)	IOLMaster 700, Mean (SD)	Anterion - IOLMaster 700 (95% CI)	*p* Value
Axial length (mm)	63	24.21 (1.45)	24.21 (1.43)	−0.01 (−0.02 to 0.01)	0.46
Anterior chamber depth (mm)	63	3.24 (0.39)	3.16 (0.40)	0.07 (0.06 to 0.08)	<0.01 *
Lens thickness (mm)	64	4.64 (0.53)	4.57 (0.53)	0.07 (0.06 to 0.08)	<0.01 *
White-to-white (mm)	64	11.68 (0.32)	11.89 (0.27)	−0.22 (−0.26 to −0.18)	<0.01 *
Central corneal thickness (μm)	64	539.33 (34.38)	542.51 (35.28)	−3.19 (−4.48 to −1.90)	<0.01 *
Pupil diameter (mm)	64	4.35 (0.91)	3.80 (1.07)	0.56 (0.38 to 0.73))	<0.01 *

Abbreviations: CI = Confidence intervals; N = number; SD = Standard deviation; μm = microns; mm = millimeters. Difference (Anterion - IOLMaster 700), 95% CI and *p* values are derived from generalized estimating equation models. Asterisk (*) indicates statistically significant differences in measurement across the two devices.

**Table 4:** Association of Keratometry Measurements with Anterion and IOL Master 700.

Corneal Curvature	Measurement Type	N (Eyes)	Anterion, Mean (SD)	IOLMaster 700, Mean (SD)	Anterion - IOLMaster 700 (95% CI)	*p* Value
Anterior Ks	K1 (D)	64	43.66 (1.41)	43.74 (1.46)	−0.08 (−0.14 to −0.02)	0.01 *
	K2 (D)	64	44.47 (1.47)	44.63 (1.53)	−0.16 (−0.23 to −0.10)	0.01 *
	Average Ks (D)	64	44.06 (1.41)	44.19 (1.44)	−0.13 (−0.19 to −0.08)	<0.01 *
	ΔK (D)	62	0.83 (0.55)	0.92 (0.63)	−0.10 (−0.16 to −0.03)	0.01 *
Posterior Ks	K1 (D)	62	−6.06 (0.25)	−5.73 (0.25)	−0.32 (−0.34 to −0.31)	<0.01 *
	K2 (D)	62	−6.36 (0.28)	−6.01 (0.28)	−0.35 (−0.37 to −0.33)	<0.01 *
	Average Ks (D)	62	−6.20 (0.26)	−5.87 (0.26)	−0.34 (−0.35 to −0.32)	0.02 *
	ΔK (D)	62	−0.30 (0.11)	−0.27 (0.11)	−0.02 (−0.04 to −0.01)	0.01 *
Total Ks	K1 (D)	63	43.13 (1.44)	43.74 (1.46)	−0.62 (−0.69 to −0.55)	<0.01 *
	K2 (D)	63	43.98 (1.46)	44.77 (1.62)	−0.79 (−0.90 to −0.68)	<0.01 *
	Average Ks (D)	63	43.55 (1.43)	44.23 (1.45)	−0.68 (−0.73 to −0.62)	<0.01 *
	ΔK (D)	63	0.85 (0.55)	0.97 (0.64)	−0.12 (−0.19 to −0.04)	<0.01 *

Abbreviations: CI = Confidence intervals; D = Diopters N = Number; SD = Standard deviation; μm = Microns; mm = Millimeters; Δ = Difference. Difference (Anterion - IOLMaster 700), 95% CI and *p* values are derived from generalized estimating equation models. Asterisk (*) indicates statistically significant differences in measurement across the two devices.

## Data Availability

The data that support the findings of this study are available from the corresponding author upon reasonable request.
